# Inhibition of p53 and/or AKT as a new therapeutic approach specifically targeting ALT cancers

**DOI:** 10.1007/s13238-019-0634-z

**Published:** 2019-05-21

**Authors:** Yuanlong Ge, Shu Wu, Zepeng Zhang, Xiaocui Li, Feng Li, Siyu Yan, Haiying Liu, Junjiu Huang, Yong Zhao

**Affiliations:** 1 MOE Key Laboratory of Gene Function and Regulation, State Key Laboratory of Biocontrol, School of Life Sciences, Sun Yat-sen University, Guangzhou, 510006 China; 2 Collaborative Innovation Center of High Performance Computing, National University of Defense Technology, Changsha, 410073 China

**Keywords:** ALT, p53, AKT, DNA damage, apoptosis, telomeres

## Abstract

While the majority of all human cancers counteract telomere shortening by expressing telomerase, ~15% of all cancers maintain telomere length by a telomerase-independent mechanism known as alternative lengthening of telomeres (ALT). Here, we show that high load of intrinsic DNA damage is present in ALT cancer cells, leading to apoptosis stress by activating p53-independent, but JNK/c-Myc-dependent apoptotic pathway. Notably, ALT cells expressing wild-type p53 show much lower apoptosis than p53-deficient ALT cells. Mechanistically, we find that intrinsic DNA damage in ALT cells induces low level of p53 that is insufficient to initiate the transcription of apoptosis-related genes, but is sufficient to stimulate the expression of key components of mTORC2 (mTOR and Rictor), which in turn leads to phosphorylation of AKT. Activated AKT (p-AKT) thereby stimulates downstream anti-apoptotic events. Therefore, p53 and AKT are the key factors that suppress spontaneous apoptosis in ALT cells. Indeed, inhibition of p53 or AKT selectively induces rapid death of ALT cells *in vitro*, and p53 inhibitor severely suppresses the growth of ALT-cell xenograft tumors in mice. These findings reveal a previously unrecognized function of p53 in anti-apoptosis and identify that the inhibition of p53 or AKT has a potential as therapeutics for specifically targeting ALT cancers.

**Electronic supplementary material:**

The online version of this article (10.1007/s13238-019-0634-z) contains supplementary material, which is available to authorized users.

## Introduction

The majority of human cancers maintain telomere length by telomerase, ~15% of all cancers, including some of the more aggressive cancers, elongate telomeres by alternative lengthening of telomeres (ALT) (Kim et al., 1994; Bryan et al., 1995). It has been reported that anti-cancer therapeutics targeting telomerase may activate ALT, inducing drug resistance (Hu et al., 2012). Therefore, cancer treatment specifically targeting ALT, although not available so far, would be very valuable.

Accumulating evidence supports that ALT cancer cells bear large amounts of intrinsic DNA damage that may cause chromatin instability (Lovejoy et al., 2012). For example, ALT cells are characterized by high frequency of telomeric homologous recombination (HR) (Dunham et al., 2000) and frequently observed DNA damage foci (53BP1 or γ-H2AX) at telomeres termed telomere dysfunction-induced foci (TIFs) (Pickett and Reddel, 2015). In addition, ALT-associated promyelocytic leukaemia (PML) bodies, which are dynamic sensor of DNA damage and cellular stress, are widely observed in ALT cancers, but not in non-ALT cells (Dunham et al., 2000; Dellaire and Bazett-Jones, 2004). With such DNA damage stress, it has been interpreted that ALT cells are competent to mount a DNA damage response (DDR).

Wild-type (wt) p53 is a tumor suppressor that, when phosphorylated and activated by ATM or ATR in cells with DNA damage, triggers downstream events leading to cell cycle arrest, cell senescence, or apoptosis (Bieging et al., 2014). Consistent with this, mutations/alterations in p53 are present in 5 to 80 percent of human cancer cells, although the strength of the association between p53 status and the cancer phenotype varies with cancer type, stage, and etiology (Petitjean et al., 2007; Bouaoun et al., 2016). P53 acts as a transcription factor through binding to DNA targets, leading to the expression of many genes that participate in a variety of biological processes including cell-cycle arrest, DNA damage repair, apoptosis, reactive oxygen species (ROS) accumulation, and so on (Schwartzenberg-Bar-Yoseph et al., 2004; Bensaad et al., 2006; Matoba et al., 2006; Lane and Levine, 2010; Hager and Gu, 2014; Ubil et al., 2014). Transactivation of genes by p53 is sensitive to both DNA sequence and p53 amount, with some sequences being responsive to much lower p53 levels than others (Inga et al., 2002; Jordan et al., 2012). For example, DNA recognition elements (REs) of cell-cycle arrest genes appear to bind to p53 with much higher affinities than most REs related to apoptosis (Weinberg et al., 2005). Therefore, it has been reported that the cellular level of p53 dictates the cell response such that lower levels of p53 result in cell cycle arrest whereas higher levels lead to apoptosis (Chen et al., 1996).

In contrast to the conventional function of p53 in pro-apoptosis, here we report that wt-p53 plays an anti-apoptotic pro-proliferative role in ALT cancer cells. ALT cells bear persistent DNA damage that induces a low-concentration of wt-p53. P53 stimulates the transcription of genes encoding mTOR and Rictor, which are key components of mTORC2 responsible for phosphorylating AKT. Activated AKT thereby suppresses the apoptosis of ALT cells. However, when a high concentration of p53 is accumulated, p53 has opposite effect by directly stimulating the transcription of apoptosis initiating genes. Identified DNA damage-p53-AKT pathway may be a new target for therapeutics of ALT cancers.

## RESULTS

### Intrinsic DNA damage, apoptotic stress and expression of p53 in ALT cells

**Figure 1:**
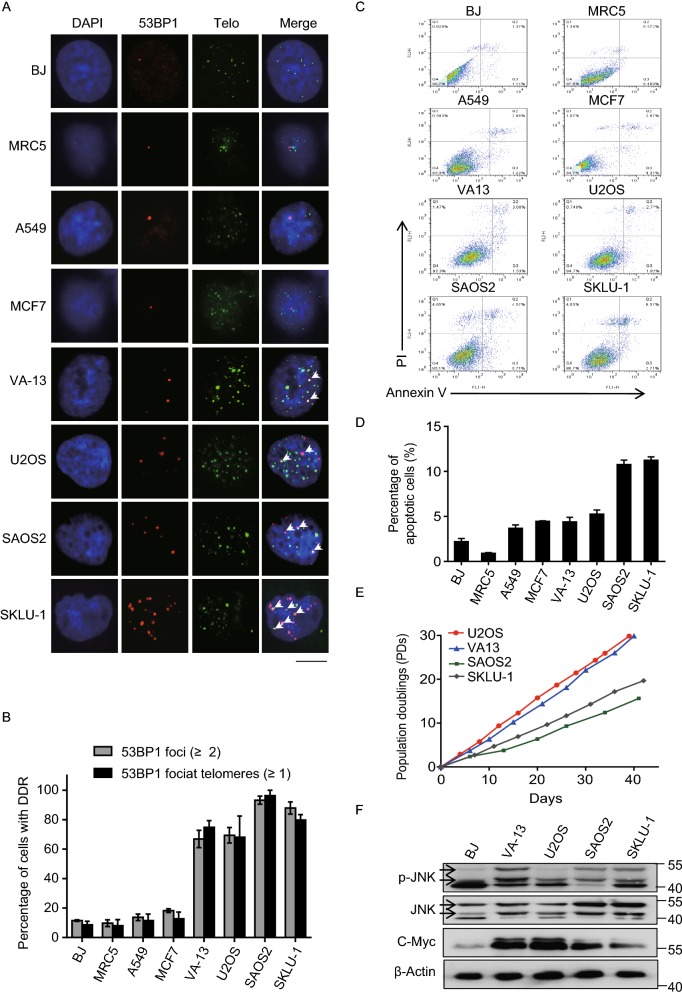
**Persistent DNA damage exists in ALT cells**. (A) IF-FISH showing DNA damage response (53BP1) in ALT (VA13, U2OS, SAOS2, SKLU-1), telomerase positive (MCF7, A549), and human normal fibroblast cells (MRC5, BJ). Scale bar: 10 μm. (B) Quantification of (A). Data represent the mean ± SEM,* n* = 3. More than 100 cells were counted for each experiment. (C) FACS analysis of apoptosis in cells listed in (A). (D) Quantification of (C). Data represent the mean ± SEM,* n* = 3. (E) Proliferation of p53-positive (VA13, U2OS) and p53-defictive (SAOS2, SKLU-1) ALT cells. (F) Western blot showing activation of JNK (phosphorylation) and accumulation of c-myc in ALT cells. Human normal BJ cells were used as a control

It was previously reported that dysfunctional telomere induced foci termed as TIF are persistently present at chromosome ends of ALT cells (Cesare et al., 2009). Here, we extended the study to examine the level of genome-wide DDR in ALT and non-ALT cells. The results showed that the abundance of 53BP1 foci, an indicator of DDR, is much higher at telomeres and genome-wide in ALT cells (VA-13, U2OS, SAOS2 and SKLU-1) than in telomerase-positive cancer (MCF7, A549) or normal human cells (MRC5, BJ Fibroblast) (Fig. 1A and 1B). Moreover, in ALT cancer U2OS cells, 53BP1 foci appear to persist throughout the cell cycle (Fig. S1A and S1B), suggesting a persistent presence of DDR in ALT cells. We also performed “constant-field gel electrophoresis assay” to detect double stranded breaks (DSBs) at telomeres (hybridized with telomeric probe) and genome (stained with gel-red). The results showed that there is no distinct difference in genomic DSBs between ALT and non-ALT cells, whereas telomeric DSBs in ALT cells are much higher than non-ALT cells (Fig. S1C), demonstrating that intrinsic DNA damage is highly associated with telomeres in ALT cells.

With a high load of persistent DDR in ALT cells, we speculated that these cells may bear higher apoptotic stress than non-ALT cells. We observed that human normal cells (MRC5 and BJ fibroblast) display the lowest apoptotic stress, as expected. Telomerase positive cancers (MCF7 and A549) have a similar apoptosis level as ALT cells that express wt-p53 (U2OS and VA13) (Fig. 1C and 1D). However, ALT cells with deficient p53 (SAOS2 and SKLU-1) contain the highest fraction of apoptotic cells (Fig. 1C and 1D). These results led to the hypothesis that p53 may play a role in suppressing apoptosis of ALT cells.

Although p53 is reported to be dysfunctional in many ALT cells (Sood et al., 2002; Mangerel et al., 2014), in checking all available ALT cancer cell lines, ~50% of ALT cancers were found to express wt-p53 (Fig. S1D). Interestingly, p53 positive VA13 and U2OS cells also proliferated faster than p53-deficient SAOS2 and SKLU-1 cells (Fig. 1E). The positive correlation between faster proliferation and expression of wt-p53 was also observed in another ALT cancer cell lines (Fig. S1E). These results suggested that wt-p53 may have an anti-apoptotic pro-proliferative function in ALT cells, which is conflict with the dogma that wt-p53 is generally a pro-apoptotic, anti-proliferative factor (Wu and Deng, 2002); thus, it is an unusual observation that we explore further below.

We then explored the mechanism by which apoptosis is induced in ALT cells. It is recognized that cancer cells can undergo both p53-dependent and p53-independent apoptosis upon severe cell stress, including DNA damage (Roos et al., 2016). JNK and c-Myc could induce apoptosis independently of p53 (Askew et al., 1991; Verheij et al., 1996). We found that JNK is activated/phosphorylated and c-Myc is accumulated not only in U2OS and VA13 cells that bear wt-p53, but also in p53-deficient SAOS2 and SKLU-1 cells, demonstrating the activation of apoptosis in ALT cells regardless of the status of p53 (Fig. 1F). In contrast, phosphorylated JNK and c-Myc accumulation were not observed in non-ALT MRC5, MCF7 and A549 cells (Fig. S1F).

### Suppression of apoptosis by p53 is specific for ALT cells

**Figure 2:**
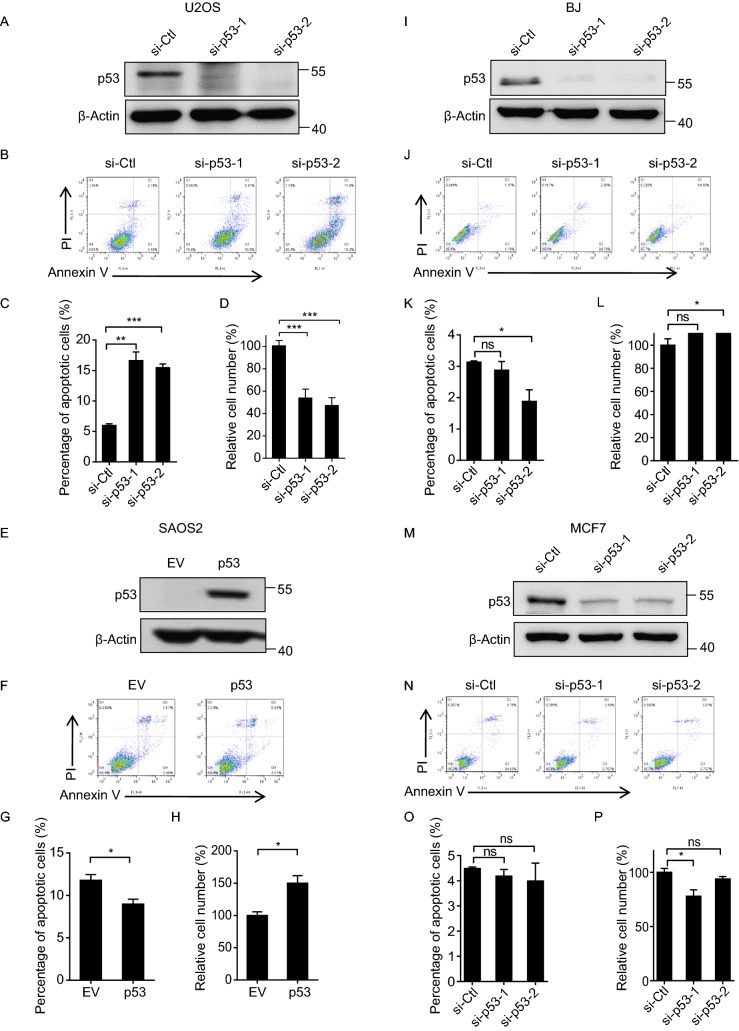
**Effect of p53 on apoptosis of ALT cells and non-ALT cells**. (A) Western blot showing depletion of p53 in U2OS cells by siRNA to p53. (B) FACS analysis of apoptotic cells in control and p53-depleted U2OS. (C) Quantification of (B). Data represent the mean ± SEM,* n* = 3. **P < 0.01, ***P < 0.001, by 2-tailed* t* test. (D) Determination of number of viable cells for U2OS with or without p53 depletion 72 h after transfection. Data represent the mean ± SEM,* n* = 6. **P < 0.01, ***P <0.001, by 2-tailed* t* test. (E) The moderately expression of wt-p53 in p53-null SAOS2 cells. (F) FACS analysis of apoptotic cells in control and p53-expressed SAOS2 cells. (G) Quantification of (F). Data represent the mean ± SEM,* n* = 3. *P < 0.05, by 2-tailed* t* test. (H) Determination of number of viable cells for SAOS2 with or without p53 expression. Data represent the mean ± SEM,* n* = 3. *P < 0.05 by 2-tailed* t* test. (I–L) same as a–d except that BJ cells were used. (M–P) same as a–d except that MCF7 cells were used

The following experiments examine whether wt-p53 modulates susceptibility of cells to apoptosis. We first tested ALT cells. In U2OS or VA13 cells, p53 was knocked-down by siRNA (Figs. 2A and S2A); this caused the fraction of apoptotic cells to increase and cell proliferation to decrease (Figs. 2B–D and S2B–D). Conversely, when wt-p53 was moderately expressed in p53 null SAOS2 cells which show a high level of apoptosis (Fig. 1C), the frequency of apoptotic cells decreased, the cell proliferation increased (Fig. 2E–H). These results suggest that wt-p53 might, in ALT cells, be an anti-apoptotic instead of a pro-apoptotic factor.

We then examined non-ALT cells. For human normal BJ and MRC5 fibroblast cells, knockdown of p53 have a limited/no effect on cell apoptosis and proliferation (Figs. 2I–L and S2E–H). Similar results were obtained for telomerase positive cancer cells MCR7 and A549 (Figs. 2M–P and S2I–L). We thus concluded that suppression of apoptosis by p53 is specifically present in ALT cells.

### Low level of p53 in ALT cells is insufficient to induce apoptosis

**Figure 3:**
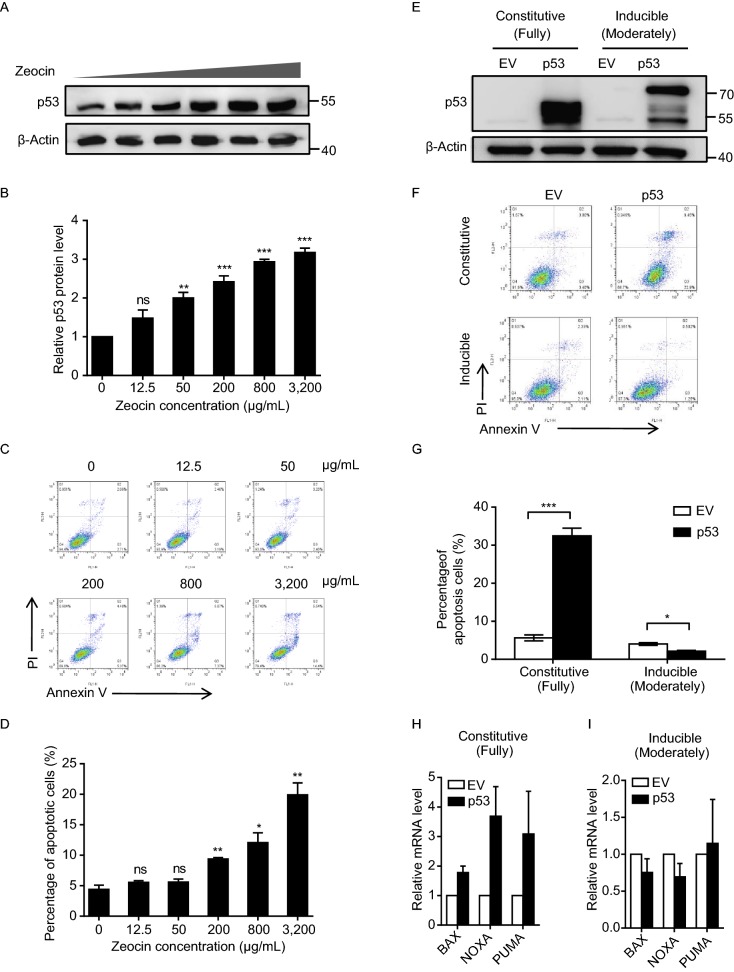
**Low level expression of p53 in ALT cells is essential for its function on anti-apoptosis**. (A) Western blot showing gradual increase of p53 level in U2OS cells treated with the increasing concentration of zeocin. (B) Quantification of (A). Untreated cells were used as a control. Data represent the mean ± SEM,* n* = 3. The t-test were performed between control and indicated concentration of zeocin. *P < 0.05, **P < 0.01, ***P < 0.001, by 2-tailed* t* test. (C) FACS analysis of apoptotic cells in U2OS treated with the increasing concentration of zeocin. (D) Quantification of (C). Data represent the mean ± SEM,* n* = 3. The* t*-test were performed between control and indicated concentration of zeocin. *P < 0.05, **P < 0.01, by 2-tailed* t* test. (E) P53 was fully (constitutive transfection) or moderately (inducible expression) expressed in U2OS. The amount of expressed p53 was determined by Western blot. (F) FACS analysis of apoptosis in U2OS cells expressing high or moderate level of p53. The expression of empty vector (EV) was used as a control. (G) Quantification of (F). Data represent the mean ± SEM,* n* = 3. *P < 0.05, ***P < 0.0001, by 2-tailed* t* test. (H) q-PCR determination of BAX, NOXA and PUMA transcript in U2OS cells expressing high or moderate level of p53. Data represent the mean ± SEM,* n* = 3

We found that in ALT U2OS cells expression level of p53 is relatively low. When U2OS cells were treated with increasing concentrations of zeocin, a radio-mimetic chemical that induces oxidative DNA damage such as ssDNA and dsDNA breaks (Chankova et al., 2007), the abundance of cellular p53 gradually increased (Fig. 3A and 3B). However, the percentage of apoptotic cells did not increase until up to 200 μg/mL of zeocin was used (Fig. 3C and 3D). These results indicated that low level of p53 is not sufficient to initiate cell apoptosis. This idea was further tested by ectopic expression of wt-p53 at variable concentration in U2OS cells. To increase the abundance of p53, the cells were transfected with constitutive or dox-inducible expression constructs for p53, generating cells with a high or moderate level of ectopic p53, respectively (Fig. 3E). The results showed that moderate expression of p53 leads to slightly decreased apoptotic cells and that the frequency of apoptosis increases significantly as high abundance of p53 is expressed (Fig. 3F and 3G). Collectively, these data demonstrated that while low level of p53 in ALT cells is anti-apoptotic, high level of p53 is pro-apoptotic. Consistently, moderately expressed p53 is insufficient to stimulate the expression of apoptosis initiating genes BAX, NOXA, and PUMA (Wu and Deng, 2002; Villunger et al., 2003), whereas fully expressed p53 was found sufficient (Fig. 3H and 3I). These results demonstrated that an abundance of p53 overrides its anti-apoptotic role in U2OS cells, and that the concentration of wt-p53 critically regulates whether its role is anti- or pro-apoptotic.

### p53-dependent activation of AKT

**Figure 4:**
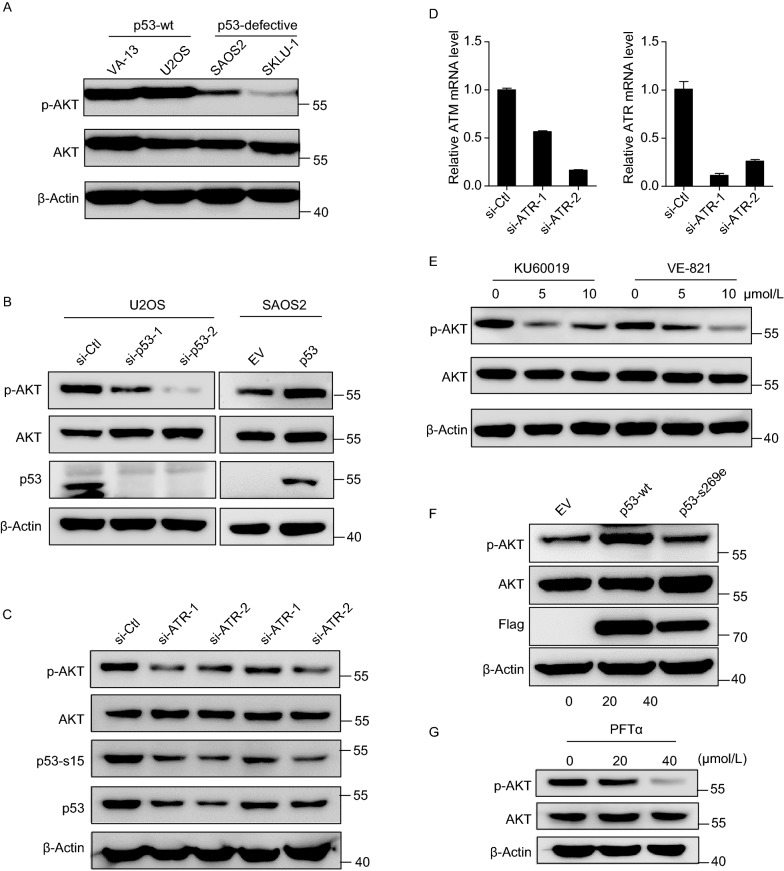
**AKT is phosphorylated in p53-dependent manner in ALT cells**. (A) Western blot determination of total and phosphorylated AKT (S473) in p53-positive (VA13, U2OS) and p53-defective (SAOS2, SKLU-1) ALT cells. (B) Knockdown of p53 in U2OS or moderate expression of p53 in SAOS2 induces down or up-regulation of p-AKT, respectively. (C) Knockdown of ATM or ATR by siRNA decreases abundance of p53, phosphorylated p53 and p-AKT. (D) Quantitative-PCR determination of the level of ATR or ATM in U2OS cells transfected with siRNA to ATR or ATM, respectively. Scramble siRNA (Si-Ctl) was used as control. Data represent the mean ± SEM,* n* = 3–4. (E) ATM (KU60019) or ATR (VE-821) inhibitor decreases abundance of p-AKT in U2OS cells. U2OS cells were treated with indicated concentration of KU60019 or VE-821 for 24 h. (F) The expression of wt-p53, but not mutant p53 (p53-s269e) defective of transcription activity, increases the level of p-AKT. (G) PFTα, an inhibitor of p53 transcription activity, suppresses the phosphorylation of AKT. U2OS cells were treated with indicated concentration of PFTα for 24 h

The AKT pathway plays an important role in regulating apoptosis and the response to cellular stress (Kennedy et al., 1997). In this regard, it is interesting to note that AKT, which is activated by phosphorylation (Chan et al., 1999), is more highly phosphorylated (p-AKT) in cells with wt-p53 (VA-13, U2OS) than in p53-defective cells (SAOS2, SKLU-1) (Fig. 4A), and in U2OS and VA13 cells, depletion of p53 decreased the abundance of p-AKT without decreasing abundance of AKT protein (Figs. 4B and S3A). Conversely, when wt-p53 was expressed in p53 null SAOS2 cells, the abundance of p-AKT increased (Fig. 4B). Given that p53 is often phosphorylated by DNA damage sensor ATM or ATR, we then examined whether ATM or ATR is required in order for p53 to stimulate phosphorylation of AKT. Indeed, the knockdown of ATR or ATM caused the amounts of p53, S15-phosphorylated-p53 (S15), and p-AKT to decrease (Fig. 4C and 4D). Similar effects were observed in U2OS cells treated with ATM or ATR inhibitors KU60019 or VE-821, respectively (Fig. 4E). These results support the hypothesis that cellular DNA damages and thereby activated ATM/ATR is required for p53-mediated phosphorylation of AKT.

Wt-p53 is a transcription factor. However, mutant p53-S269E is a dominant-negative p53 variant that lacks the transcriptional activity (Wu et al., 2011). Here, we observed that while expression of wt-p53 slightly increased the level of p-AKT, expression of p53-S269E decreased p-AKT level, suggesting that transcription activity of p53 is required for phosphorylation of AKT (Fig. 4F). Consistently, the exposure of cells to PFTα, an inhibitor of the transcription activation activity of p53 (Komarov et al., 1999), decreased the abundance of p-AKT in U2OS and VA13 cells (Figs. 4G and S3B).

### p53 stimulates the expression of mTORC2 that is responsible for phosphorylation of AKT

**Figure 5:**
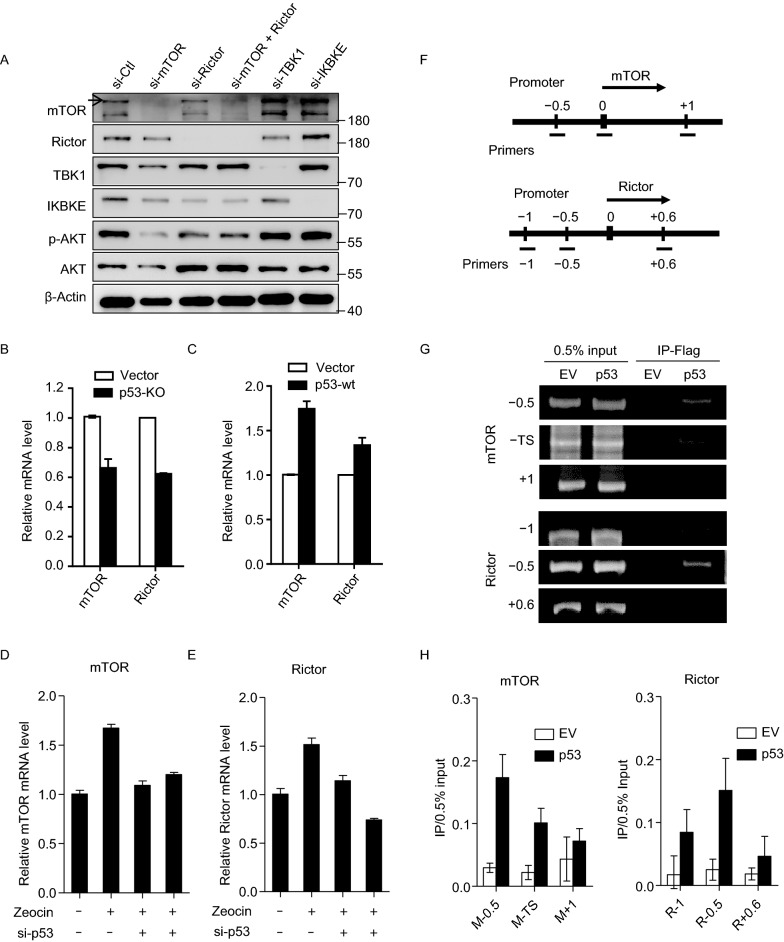
**P53 induces p-AKT by promoting the transcription of mTOR and Rictor**. (A) Knockdown of mTOR and/or Rictor, but not TBK1 or IKBKE, decreases abundance of p-AKT in U2OS cells. (B) Knockout of p53 induces decreased expression of mTOR and Rictor. Abundance of mTOR and Rictor mRNA was determined by q-PCR in U2OS with or without depletion of p53. Data represent the mean ± SEM,* n* = 3. (C) Expression of p53 in SAOS2 cells increases the level of mTOR and Rictor mRNA. Abundance of mTOR and Rictor mRNA was determined by q-PCR. Data represent the mean ± SEM,* n* = 4. (D) Zeocin stimulates transcription of mTOR in p53 dependent manner. U2OS cells transfected with siRNA to p53 were treated with zeocin (200 μg/mL) for 6 h and abundance of mTOR was determined by q-PCR. Data represent the mean ± SEM,* n* = 4. (E) Same as (D) except that the abundance of Rictor was determined. Data represent the mean ± SEM,* n* = 4. (F–H) ChIP assay to determine the association between p53 with promoter of mTOR and Rictor gene. Flag-p53 was expressed in U2OS for ChIP assay. Primers were designed to cover 1kb region surrounding the transcription start site (TSS) of mTOR and Rictor gene. Data represent the mean ± SEM,* n* = 3

AKT is reported to be phosphorylated by several kinases including IKBKE, TBK1 and the mTORC2 complex that consists of mTOR and Rictor (Sarbassov et al., 2006; Ou et al., 2011; Mahajan and Mahajan, 2012). Strikingly, depletion of mTOR or Rictor in U2OS cells, but not of TBK1 or IKBKE, reduced the abundance of p-AKT (Fig. 5A), suggesting that AKT is phosphorylated primarily by the mTORC2 complex. This was confirmed by treating cells with mTORC1/C2 inhibitor KU0063794, which suppressed phosphorylation of AKT, while rapamycin, a specific mTORC1 inhibitor, did not (Fig. S4A). This suggested that activated p53 might stimulate expression of mTOR or Rictor. Consistent with this, depletion of p53 in U2OS cells or overexpression of wt-p53 in SAOS2 cells led to decreased or increased expression of mTOR and Rictor, respectively (Fig. 5B and 5C). In addition, when U2OS cells were treated with low dose of zeocin, the abundance of p53 and the expression level of mTOR and Rictor increased, and the increase of mTOR and Rictor transcripts was suppressed by knockdown of p53 (Figs. 5D,5E and S4B). These results demonstrated that DNA damage-induced increase of mTOR and Rictor transcription is mediated by p53.

P53 serving as a transcription factor of mTOR and Rictor was further examined by a standard ChIP assay, which demonstrated a physical interaction between p53 and targeted genes. Three pairs of primers were designed to detect the presence of p53 on DNA over 1 kb region surrounding the transcription start site (TSS) of mTOR and Rictor gene (Fig. 5F). ChIP assay showed that p53 primarily associates with a 0.5 kb upstream promoter region near the transcription start site of mTOR and Rictor gene (Fig. 5G and 5H).

### Inhibition of p53 and/or AKT induces rapid death of ALT cancer cells

**Figure 6:**
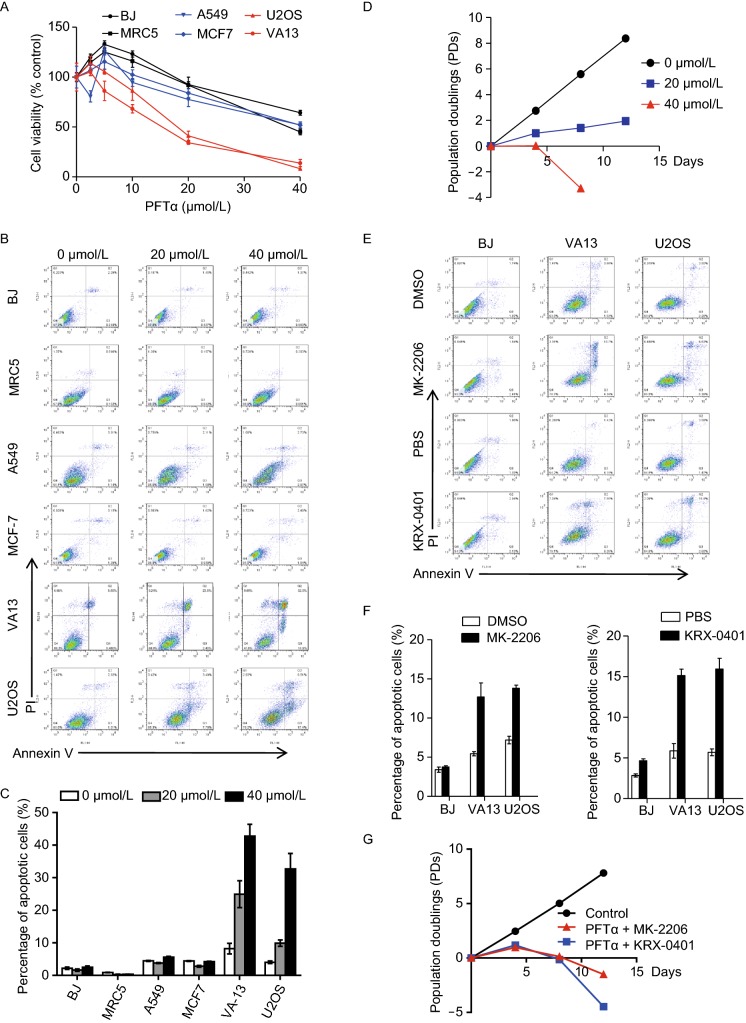
**p53 and/or AKT as a therapeutic target in ALT cancer cells**. (A) Cell viability analysis of normal human BJ, MRC5 cells, telomerase-positive A549, MCF7 cells and ALT positive U2OS, VA13 cells that are treated with the increasing dose of p53 inhibitor PFTα. (B) FACS analysis of apoptosis in indicated cells treated with 20 or 40 μmol/L of PFTα for 3 days. PBS treated cells were used as a control. (C) Quantification of (B). Data represent the mean ± SEM,* n* = 3–4. (D) Proliferation of U2OS cells treated with 20 or 40 μmol/L PFTα. (E) FACS analysis of apoptosis in BJ, VA13 and U2OS cells treated with MK-2206 (2.5 μmol/L) or KRX-0401(25 μmol/L) for 3 days. (F) Quantification of (E). Data represent the mean ± SEM,* n* = 3–5. (G) Proliferation of U2OS cells treated with 20 μmol/L PFTα in combination with MK-2206 (2.5 μmol/L) or KRX-0401 (25 μmol/L)

Results presented above suggested that ALT cells with wt-p53 experience p-AKT-mediated suppression of apoptosis. Therefore, we speculated that it might be possible to stimulate apoptosis in ALT cells by inhibiting p53 and/or phosphorylation of AKT. To this end, p53 inhibitor PFTα was used to treat human normal cells (BJ, MRC5), non-ALT cancer cells (A549, MCF7) and ALT cells expressing wt-p53 (U2OS, VA13). We found that U2OS and VA13 cells are hypersensitive to PFTα because their cell viability dramatically declines upon the treatment (Fig. 6A). Consistently, PFTα treatment remarkably increased apoptosis of U2OS and VA13 cells, with a limited effect on human normal cells and non-ALT cancer cells (Fig. 6B and 6C). Moreover, treatment with PFTα significantly slowed proliferation of U2OS cells at a low dose (20 μmol/L) and resulted in mass cell death at a high dose (40 μmol/L) (Fig. 6D).

Similar to the effect induced by PFTα, the allosteric AKT inhibitors MK-2206 increased apoptosis significantly in VA13 and U2OS cells, but not in BJ cells (Figs. 6E, 6F and S5A). Other AKT inhibitor KRX-0401 slightly increased apoptosis of BJ cells, but dramatically increased apoptosis of VA13 and U2OS cells (Figs. 6E, 6F and S5A). Moreover, cancer cells treated with a low dose of PFTα (20 μmol/L) in combination with AKT inhibitor (MK-2206 or KRX-0401) rapidly lost viability (Fig. 6G).

Interestingly, p53-negative ALT cancers are also hypersensitive to and rapidly undergo apoptosis in the presence of MK-2206 or KRX-0401 (Fig. S5B–D). KRX-0401 and MK-2206 induced mass death of SAOS2 and SKLU-1, respectively (Fig. S5E). We reasoned that p-AKT, which is very limited in number in p53-deficient ALT cells, is absolutely required to suppress apoptosis induced by intrinsic DNA damage (Fig. 4A). Therefore, agents targeting the AKT pathway have a potential for therapeutics of all ALT cancers, independent of their p53 status.

### The growth of ALT positive xenograft tumors in mice treated with p53 inhibitor

**Figure 7:**
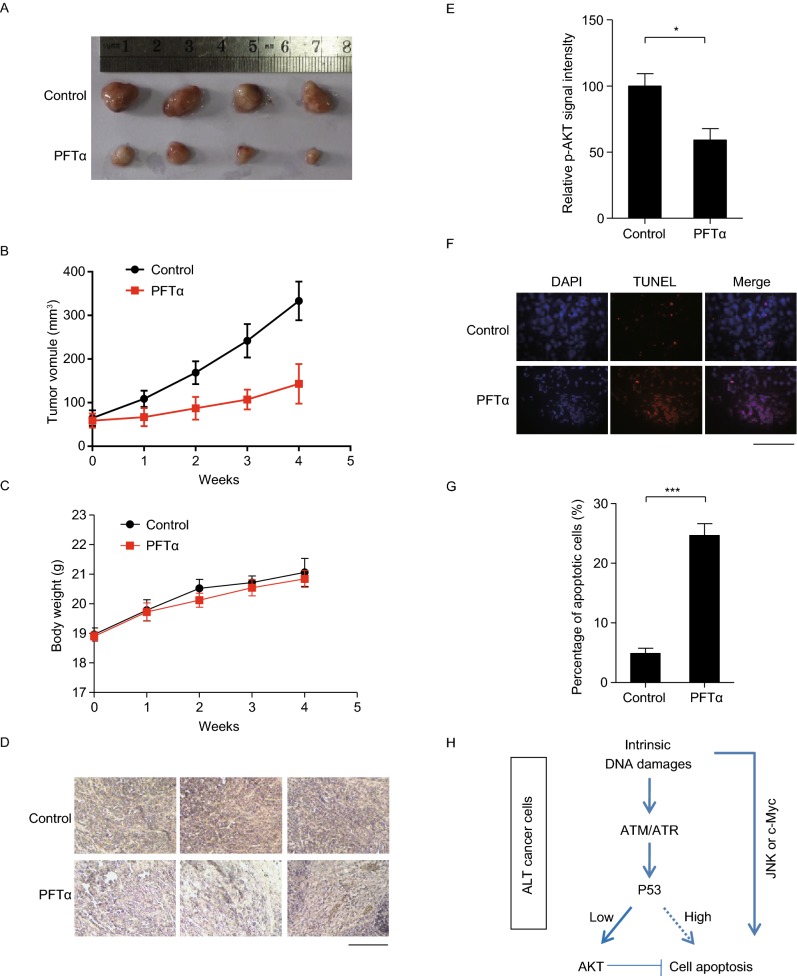
**P53 inhibitor PFTα suppresses the growth of ALT cancer xenograft tumors in mice by inducing cell apoptosis**. (A) Mice (female, four-week-old) were injected (i.p.) with inoculum containing 3 × 10^6^ U2OS cells. After 40 days when xenograft tumors were visible, 2.2 mg/kg of PFTα dissolved in phosphate-buffered saline (PBS) was injected (intraperitoneal injection) two times a week for 4 weeks. For the control group, the same volume of PBS was injected. Representative images of xenograft tumors were showed (*n* = 4 per group). (B) Quantification of data in (A). Data represent the mean ± SEM. (C) The body weight of the mice was measured over the treatment period. Data represent the mean ± SEM. (D) Representative histology staining images showing abundance of p-AKT in cells of xenograft tumor tissue from control and PFTα-treated mice. Scale bar: 500 μm. (E) Quantification of (D). Data represent the mean ± SEM,* n* = 3. *P < 0.05, by 2-tailed* t* test. (F) Representative histology staining images showing the percentage of apoptotic cells of xenograft tumor tissue from control and PFTα-treated mice. Scale bar: 100 μm. (G) Quantification of (F). For each group, 1,000 or more cells were examined. Data represent the mean ± SEM,* n* = 11, **P < 0.001, by 2-tailed* t* test. (H) Working model

We next investigated the effects of p53 inhibitor PFTα on cancer cells *in vivo* using a murine xenograft model, where the xenograft inoculum consisted of ALT-positive U2OS cells. First, we examined how PFTα affects the formation of xenograft tumor in mice. Immediately after injection with U2OS cell inoculum and two times a week thereafter, mice were injected (Intraperitoneal injection) with PFTα (2.2 mg/kg). After 4 weeks, we observed xenograft tumor in untreated mice (four of four cases), but only one of four PFTα treated mice showed detectable tumor (Fig. S6A). Next, we explored whether inhibition of p53 is able to suppress the growth of xenograft tumor *in vivo*. To this end, PFTα treatment was not initiated until xenograft tumor was formed in mice (40 days after U2OS cell inoculum), and the size of these tumors was determined over next four weeks of treatment. The results showed that although tumors were still visible after 4 weeks in the treated group, the tumor size was much smaller than that in the untreated group (Fig. 7A and 7B). PFTα did not affect the body weight of mice inoculated with U2OS cells (Figs. 7C and S6B). Moreover, the level of p-AKT in xenograft tumor cells in PFTα treated mice was much lower than that in untreated mice (Fig. 7D and 7E), suggesting that activation of AKT is inhibited by PFTα *in vivo*. In addition, the relative amount of apoptotic cells in xenograft tumor in mice treated with PFTα was also higher than that in untreated mice (Fig. 7F and 7G), demonstrating the inhibition of p53 by PFTα resulted in apoptosis of U2OS cells *in vivo*. Altogether, these results indicated that PFTα inhibits the growth of ALT-positive U2OS xenograft tumors in mice.

## DISCUSSION

The pro-apoptotic function of p53 is well recognized. However, it has been recently recognized that wt-p53 suppresses metabolic stress-induced ferroptosis (non-apoptotic iron-dependent cell death), suggesting that wt-p53, under specific circumstances, may function to help cancers to cope with cellular stress (Tarangelo et al., 2018). Recently, Jinchul Kim et al. reported that wt-p53 plays an oncogenic role by suppressing oxidative phosphorylation in hepatocarcinoma (HCC) (Kim et al., 2019). It is certainly possible that wt-p53 may also promote the metabolic switch from oxidative phosphorylation to glycolysis in ALT cancer cells as it does in HCCs. This, however, does not explain how ALT cancers overcome high stress of apoptosis induced by intrinsic telomeric DNA damage. Here, our study documents a previously unrecognized p-AKT-mediated anti-apoptotic function of wt-p53 in ALT cancer cells. We suggest that in these cells, on account of high load of DNA damage, ALT cells suffer from high apoptotic stress through activating p53 independent pathways such as JNK or c-Myc; meanwhile, intrinsic DNA damage leads to low level of p53 that suppresses apoptosis through activation/phosphorylation of AKT; however, high level of p53 induced by either exogenously imposed DNA damage or exotic expression of p53 would result in apoptosis of ALT cells (Fig. 7H).

### Anti-apoptosis activity of p53 specifically exists in ALT cells

It has been previously reported that p53-deficient mouse fibroblasts are more sensitive to genotoxins and DSB-triggered apoptosis than p53 wild-type cells (Lackinger et al., 2001; Lips and Kaina, 2001), raising the possibility that in specific cell systems and/or under given circumstances, p53 may function to protect against DSB induced apoptosis. In accord with this, our results showed that although low level of p53 in human ALT cells is insufficient to activate the expression of apoptosis-initiating genes such as BAX, NOXA and PUMA (Fig. 3H and 3I), it is adequate for stimulating the transcription of mTOR and Rictor (Fig. 5B and 5C), thus resulting in activation/phosphorylation of AKT that acts to suppress DNA damage-induced apoptosis. We thus proposed that the anti-apoptotic function of p53 represents a new mechanism for ALT cells to counteract intrinsic apoptotic stress. This mechanism may also exist in mice because mice cells are ALT positive (Neumann et al., 2013). However, in telomerase positive cancer and human normal cells, p53 does not display anti-apoptosis activity (Figs. 2I–P and S2E–L).

P53 is a short-lived protein that is maintained at low, often undetectable, levels in normal cells. In response to an activating signal, such as DNA damage, p53 protein is stabilized by ATM/ATR mediated phosphorylation and its level increases. The function of p53 is dependent on both amount and type of DNA damage (Lassus et al., 1996; Roos and Kaina, 2006). There are two unique features of ALT cells that may dictate anti-apoptosis function of p53. First, ALT cells bear persistent apoptotic stress in p53-indepednent manner. We observed that apoptosis exists not only in p53-positive U2OS and VA13 cells, but also in p53-deficient SAOS2 and SKLU-1 cells (Fig. 1C and 1D), and that both JNK and C-myc pathways are activated in these cells that could lead to cell apoptosis independently of p53 (Fig. 1F). Second, DNA damage in ALT cells stimulates consistent but relative low level of p53 that is not enough to initiate p53-mediated apoptosis (Fig. 3A–D). The nature of DNA damages in ALT cells is still not fully understood. Increasing evidence suggests that the defect in DNA replication machinery in ALT cells may cause incompletely replicated DNA that induces persistent DDR (Clynes et al., 2015; Dilley et al., 2016; Roumelioti et al., 2016). Whether similar DDR is present in other cell type and is capable to stimulate a pro-apoptotic function of p53 remains for further investigation.

### P53 activates AKT that prevent cells from undergoing apoptosis

Our data show that p53 activates AKT by increasing the expression of mTORC2 component mTOR and Rictor (Fig. 5B and 5C). Activated AKT could prevent DNA damage-induced apoptosis in two ways. On the one hand, p-AKT could directly phosphorylate apoptosis execution factors (BAD and caspase-9) and suppress their function (del Peso et al., 1997; Cardone et al., 1998). Alternatively, p-AKT could activate NF-κB, thereby stimulating the transcription of anti-apoptotic BCL-2 family proteins BFL1/A1, BCL-XL and neuroligin 3 (NRL3) (Pahl, 1999). On the other hand, p-AKT could upregulate the expression of MRE11 through activating GSK3β/β-catenin pathway (Deng et al., 2011). Therefore, increased capacity for DNA damage repair relieves apoptotic stress.

It has been previously reported that p-AKT binds to, and phosphorylates MDM2, thereby increasing ubiquitin ligase activity of MDM2 and promoting its import into nucleus (Mayo and Donner, 2001). Because MDM2 plays an essential role in inactivation/degradation of p53, increased amount of MDM2 in nucleus would result in decrease of p53 (Zhou et al., 2001). As such, in ALT cells AKT and p53 may form a negative regulation mechanism that keeps p53 at a relatively low level: intrinsic DNA damage induces p53 → p53 activates AKT → p-AKT stimulates MDM2 → MDM2 reduces the amount of p53.

### P53 and AKT as a target for ALT cancer therapy

Because of its near universal alteration in cancer, p53 is an attractive target for the development of new cancer therapeutics. Current strategy focuses on increasing the level of wt-p53 in cancer cells by either restoring wild-type properties to mutant p53 or preventing the binding of MDM2/MDM4 to wt-p53, thereby blocking its degradation (Duffy et al., 2014). In the context of ALT cancers, potential side effects from the use of these treatments should be taken into consideration. Under certain conditions, p53 may help cancer cells to escape the apoptosis induced by chemotherapy or radiotherapy.

Because ALT cancers are known to be aggressive and difficult to treat and conventional anti-cancer therapeutics are known to convert some telomerase-positive cells to ALT cells (Hu et al., 2012), effective therapeutics for ALT cancers, although not yet available, are urgently needed and would be extremely valuable. The discovery of DNA damage-p53-AKT pathway provides strong rationale for treating ALT cancers that express wt-p53 with agents that inhibit p53 and/or AKT without concomitant exposure to DNA damaging agents (Fig. 6). In addition, because of high apoptosis stress induced by persistent DNA damage, p53 deficient ALT cancers are also hypersensitive to suppression of AKT, suggesting that inhibitors of AKT may be especially beneficial in treatment of these ALT cancers (Fig. S5C–E). Further studies are needed to explore the clinical potential of these treatments.

## MATERIALS AND METHODS

### Cell culture, plasmids and compounds

U2OS, VA13, SAOS2, SKLU-1, A549, MCF-7, MRC5, BJ cells were obtained from the Cell Resource Center of Peking Union Medical College and were grown in DMEM (Gibco) with 10% FBS (Gibco) and cultured at 37 °C under 5% CO_2_. All cell lines were negative for mycoplasma contamination and they were authenticated using STR (Short Tandem Repeat) profiling method. Wild-type p53 gene was amplified from HEK293 mRNA and cloned into Phage-tet-SFB (moderate expression) and pLenti-HA/Flag (full expression). The mutant (S269E) of p53 was constructed. KU60019, VE-821, Pifithrin-α (PFTα), MK-2206, KRX-0401, rapamycin, KU0063794 were purchased from Selleck. Zeocin was purchased from ThermoFisher.

### Gene silencing and knockout

SiRNA was transfected into target cells in a 6-well plate using Lipofectamine® RNAiMAX Transfection Reagent (Invitrogen), according to the manufacturer’s instructions. siRNA against p53 (si-1: 5′-GCAUGAACCGGAGGCCCAUdTdT-3′; si-2: 5′-GACUCCAGUGGUAAUCUACdTdT-3′), mTOR (5′-GTAAATGCTTCCACTAAACdTdT-3′), Rictor (5′-GCAGCCUUGAACUGUUUAAdTdT-3′), TBK1 (5′-UGACAGAGAUUUACUAUCAdTdT-3′), IKBKE (5′-GCUGAACCACCAGAACAUUdTdT-3′) were provided by Suzhou GenePharma Co., Ltd. The scrambled sequence was used as a control. For p53 gene knockout, the sgRNA sequence (5′-CGCTATCTGAGCAGCGCTCA-3′ and 5′-CCATTGTTCAATATCGTCCG-3′) were cloned into lenti-CRISPRv2 consisting of Flag-Cas9. Lentivirus was packaged into 293T cells using calcium phosphate transfection. Viral supernatants were collected to infect target cells. Lenti-CRISPRv2 with scrambled sequence was used as a control.

### Western blot

Cells were directly lysed in 2× SDS loading buffer and boiled for 15 min. Proteins were separated by SDS–PAGE, transferred to PVDF membrane, and probed with antibodies specific for, p-AKT (1:2000 dilution, s473, 4060, Cell Signaling Technology), AKT (1:2000 dilution, ab32505, Abcam), p-p53 (1:2000 dilution, s15, 9284, Cell Signaling Technology), p-JNK (1:2000 dilution, 9251, Cell Signaling Technology), JNK (1:2000 dilution, 9252, Cell Signaling Technology), c-Myc (1:2000 dilution, ab32072, Abcam), p53 (1:1000 dilution, sc-126, Santa Cruz), and Flag (1:5000 dilution, F1804, Sigma), mTOR (1:2000 dilution, 2983, Cell Signaling Technology), Rictor (1:2000 dilution, 2114, Cell Signaling Technology), TBK1 (1:2000 dilution, 3504, Cell Signaling Technology), IKBKE (1:2000 dilution, 3416, Cell Signaling Technology). β-Actin (1:5000 dilution, 66009-1-Ig, Proteintech) antibody was used as a loading control. Samples derived from the same experiment and gels/blots were processed in parallel.

### Cell cycle synchronization

U2OS cells were synchronized with thymidine (2 mmol/L) for 19 h, washed with pre-warmed PBS (3 times), then released into fresh medium for 9 h; thymine (2 mmol/L) was then added for 17 h followed by washing with pre-warmed PBS (3 times) before release into fresh medium for 0, 3, 6 or 9 h.

### Immunofluorescence and fluorescence *in situ* hybridization (IF-FISH)

Briefly, cells on the coverslip were fixed with 4% paraformaldehyde, and then permeabilized with 0.5% Triton X-100 (in 1× PBS). Cells were incubated overnight at 4 °C with primary antibodies against 53BP1 (1:2000 dilution, NB100-304, Novus), washed three times with 1× PBST, and incubated with secondary antibodies (1:2000 dilution, DyLight 549-conjugated anti-mouse, KPL). Cells were washed three times with 1× PBST, re-fixed with 4% paraformaldehyde for 30 min, dehydrated by ethanol series solution, denatured at 85 °C for 5 min, and then hybridized with FITC-labeled (CCCTAA)_3_ PNA probe (Panagene) at 37 °C for 4 h. The cells were washed and mounted with DAPI. Fluorescence was detected and imaged using Zeiss Axion Imager Z1 microscope.

### Quantitative real-time PCR

Total RNA was extracted from cells using RNAiso Plus Reagent (Takara) according to manufacturer’s instructions. 1.0 μg of total RNA was reverse-transcribed to cDNA using PrimeScript RT reagent Kit (Takara). cDNA was used for real-time PCR using 2× RealStar Green Fast Mixture (GenStar). β-Actin was used as internal control for all experiments. The following primers were used for amplification: β-actin-forward: 5′-CATGTACGTTGCTATCCAGGC-3′; β-actin-reverse: 5′-CTCCTTAATGTCACGCACGAT-3′; BAX-forward: 5′-CCCGAGAGGTCTTTTTCCGAG-3′; BAX-reverse: 5′-CCAGCCCATGATGGTTCTGAT-3′; NOXA-forward: 5′-ATGCTGCGTTTCACCAGGG-3′; NOXA-reverse: 5′-TCCATGCTACTTGCACTTGTTCCT-3′; PUMA-forward: 5′-GACCTCAACGCACAGTACGAG-3′; PUMA-reverse: 5′-AGGAGTCCCATGATGAGATTGT-3′; mTOR-forward: 5′-GCAGATTTGCCAACTATCTTCGG-3′; mTOR-reverse: 5′-CAGCGGTAAAAGTGTCCCCTG-3′; Rictor-forward: 5′-AGAAGCACGATTTCTAGCCAGT-3′; Rictor-reverse: 5′-AGTAGACCTCGCCTTATTTCCA-3′.

### Chromatin immunoprecipitation

U2OS stably expressing p53 were cross-linked with 1% formaldehyde for 10 min at room temperature. Reaction was terminated by 1.25 mmol/L Glycine, and cells were washed twice with cold PBS, re-suspended in SDS lysis buffer (50 mmol/L Tris-HCl, pH = 8.1, 10 mmol/L EDTA, 1% SDS) and sonicated until DNA fragmentation reached sizes of 200 bp to 1 kb. Chromatin immunoprecipitation (ChIP) was carried out overnight at 4 °C with flag-beads (M8823, sigma). Beads were washed three times, and eluted with 0.1 mol/L NaHCO_3_ & 1% SDS, followed by reverse cross-linking and phenol-chloroform extraction. DNA fragments were precipitated by ethanol. The following primers were used to identify fragments of mTOR and Rictor promoters: M-0.5-forward: 5′-CTGCATCAAAAGACGTTTCGGGCT-3′; M-0.5-reverse: 5′-ACTTGGCTATGGTTTCTCTGGAGTG-3′; M-TS-forward: 5′-TTCGGCCAAGGCCTCAGCTG-3′; M-TS-reverse: 5′-GCTTCTCTTCTTCATTACTATTGGCC-3′; M+1-forward: 5′-GACAGAGCGAAACTCCGTCTCA-3′; M+1-reverse: 5′-CCAGGTTTTTCCTCTGTGCCTTAC-3′; R−1-forward: 5′-TCCAGGGCACTTACTCATCCAACA-3′; R−1-reverse: 5′-ACTGACCCAGCAGCTTTCTCTTGA-3′; R−0.5-forward: 5′-TCTATGGCAGGGCTTCAGAGCAA-3′; R−0.5-reverse: 5′-AGTTCCCACGAGGAAAGTCCCATT-3′; R+0.6-forward: 5′-TGCAGGAGGATGTTTGAGGGAAGA-3′; R+0.6-reverse: 5′-AAAGGGAAGCAGAAGGGAAACAGC-3′.

### Cell apoptosis assay

To detect apoptotic cells, cells treated with siRNA or indicated concentrations of compounds were co-stained with Annexin-V (FTIC) and PI (Annexin-V/PI apoptosis detection kit, KGA105, KeyGen BioTech) according to manufacturer’s instructions. Stained cells were analyzed by FACS Calibur (BD Bioscience) and data were analyzed with FlowJo software.

### Constant-field gel electrophoresis (CFGE)

CFGE assay was performed as described previously (Mao et al., 2016). Cells were imbedded in 0.7% agarose, lysed with 0.5% SDS in Tris-HCl and digested with RNase A (100 μg/mL) and proteinase K (250 μg/mL) at 37 °C overnight. Gel electrophoresis was performed using 0.7% agarose in TAE buffer. Genomic DNA was stained by gel-red, and telomeric DNA was detected by hybridization with C-rich probe.

### Establishment of xenograft models using human U2OS cells

A total of 3.0 × 10^6^ U2OS cells were inoculated subcutaneously into the dorsal flank of the four-week-old female BALB/c nude mice in 100 μL PBS. Mice were randomly divided into control, and PFTα treatment groups. 2.2 mg/kg of PFTα dissolved in phosphate-buffered saline (PBS) was injected (Intraperitoneal injection) two times a week for 4 weeks. No animal died during the experimental period. The tumor size was estimated from the measurements of the longest diameter across the tumor and the corresponding perpendicular diameter.

### Histology staining of p-AKT and apoptotic cells in xenograft tumor

Tumors were fixed overnight in 4% paraformaldehyde at room temperature, transferred to 70% ethanol and embedded in paraffin. 4 μm sections on slides were dewaxed in xylene, and then sequentially rehydrated in 100%, 95%, 70% ethanol and PBS buffer. For immunohistochemistry, sections were blocked with 5% goat serum in PBS buffer, then incubated overnight with primary antibodies against p-AKT (1:1000 dilution, s473, 4060, Cell Signaling Technology) at 4 °C, washed three times with 1× PBST, and incubated with secondary antibodies (HRP-conjugated anti-rabbit, KPL). Sections were washed three times with 1× PBST and stained with DAB and hematoxylin (for DNA staining) according to standard protocols. To assess the frequency of apoptosis, tissue was analyzed using a TdT-mediated dUTP-digoxigenin nick end labeling (TUNEL) method. Staining was performed according to manufacturer’s instructions (TUNEL assay Kit, KGA7061, KeyGEN BioTECH).

### Statistical analysis

Statistical analysis was performed using GraphPad Prism version 5 (GraphPad Software). Data are expressed as the mean ± SEM. Comparisons between groups were made using a 2-tailed unpaired Student’s *t* test. A *P* value of less than 0.05 was considered statistically significant.

### Study approval

All animal experiments were approved by the Institutional Animal Care and Use Committee of Sun Yat-sen University (Guangzhou, China).

## Electronic supplementary material

Below is the link to the electronic supplementary material.

Supplementary material 1 (PDF 1852 kb)
