# Correction to: Inhibition of p53 and/or AKT as a new therapeutic approach specifically targeting ALT cancers

**DOI:** 10.1007/s13238-019-0640-1

**Published:** 2019-06-12

**Authors:** Yuanlong Ge, Shu Wu, Zepeng Zhang, Xiaocui Li, Feng Li, Siyu Yan, Haiying Liu, Junjiu Huang, Yong Zhao

**Affiliations:** 1 MOE Key Laboratory of Gene Function and Regulation, State Key Laboratory of Biocontrol, School of Life Sciences, Sun Yat-sen University, Guangzhou, 510006 China; 2 Collaborative Innovation Center of High Performance Computing, National University of Defense Technology, Changsha, 410073 China

## Correction to: Protein Cell 10.1007/s13238-019-0634-z

In the original publication the labels in Fig. 4C and 4D are incorrectly published. The correct labels for Fig. 4C and 4D is provided in this correction.

**Figure 4:**
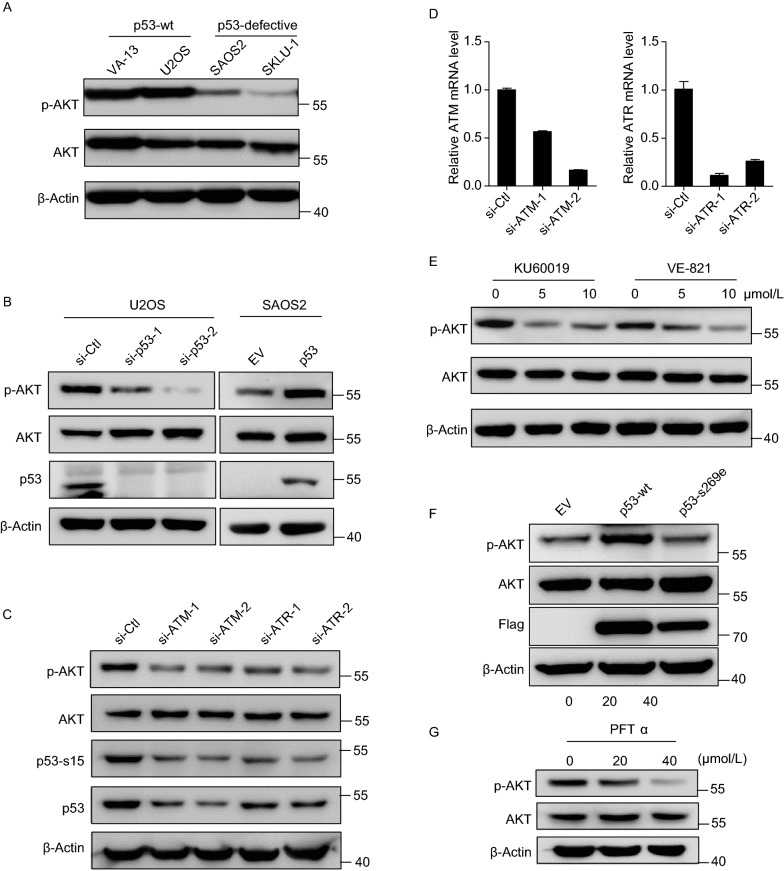
**AKT is phosphorylated in p53-dependent manner in ALT cells**. (A) Western blot determination of total and phosphorylated AKT (S473) in p53-positive (VA13, U2OS) and p53-defective (SAOS2, SKLU-1) ALT cells. (B) Knockdown of p53 in U2OS or moderate expression of p53 in SAOS2 induces down or up-regulation of p-AKT, respectively. (C) Knockdown of ATM or ATR by siRNA decreases abundance of p53, phosphorylated p53 and p-AKT. (D) Quantitative-PCR determination of the level of ATR or ATM in U2OS cells transfected with siRNA to ATR or ATM, respectively. Scramble siRNA (Si-Ctl) was used as control. Data represent the mean ± SEM, n = 3-4. (E) ATM (KU60019) or ATR (VE-821) inhibitor decreases abundance of p-AKT in U2OS cells. U2OS cells were treated with indicated concentration of KU60019 or VE-821 for 24 h. (F) The expression of wt-p53, but not mutant p53 (p53-s269e) defective of transcription activity, increases the level of p-AKT. (G) PFTα,an inhibitor of p53 transcription activity, suppresses the phosphorylation of AKT. U2OS cells were treated with indicated concentration of PFTα for 24 h

