# Supplementary material for: Inhibition of p53 and/or AKT as a new therapeutic approach specifically targeting ALT cancers
Source: Protein Cell. 2019 May 21;10(11):808–24. doi: 10.1007/s13238-019-0634-z (PMC6834538; doi:10.1007/s13238-019-0634-z)
Supplement: Supplementary file 1 — Supplementary material 1 (PDF 1852 kb) [file 13238_2019_634_MOESM1_ESM.pdf]

SUPPLEMENTARY INFORMATION

Inhibition of p53 and/or AKT as a New Therapeutic Approach Specifically Targeting ALT Cancers

Yuanlong Ge[1,2,*], Shu Wu[1,2,*], Zepeng Zhang[1,2], Xiaocui Li[1,2], Feng Li[1], Siyu Yan[1], Haiying Liu[1,2], Junjiu Huang[1], Yong Zhao[1,2]

[1]MOE Key Laboratory of Gene Function and Regulation, State Key Laboratory of Biocontrol, School of Life Sciences, Sun Yat-sen University, Guangzhou, 510006, P. R.China.

[2]Collaborative Innovation Center of High Performance Computing, National University of Defense Technology, Changsha 410073, P. R. China.

[*]These authors contributed equally to this work.

Correspondence: Yong Zhao, E-mail: zhaoy82@mail.sysu.edu.cn, Tel: 86-203-994-3401, Fax: 86-203-994-3778

Supplementary Figures

Figure S1

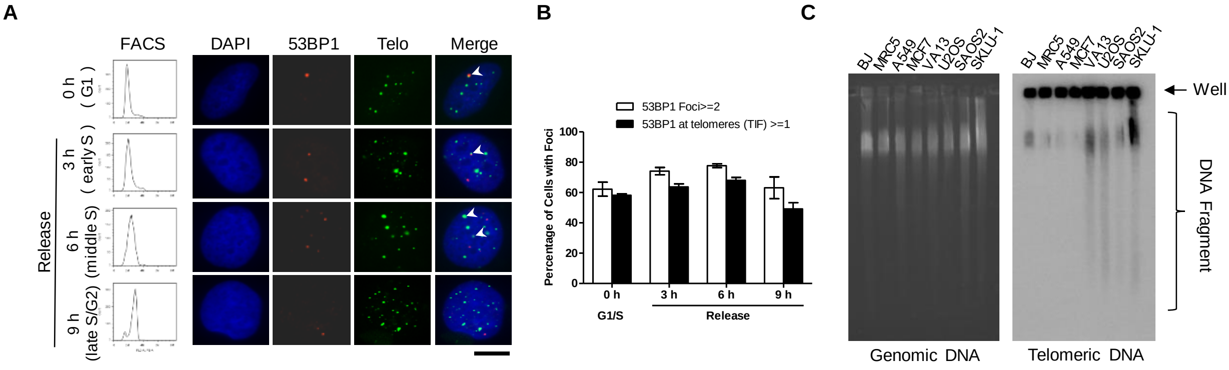

D

Cancer Cell Lines	p53	Cancer Type
U2OS	WT	Osteosarcoma
CAL-72	WT	Osteosarcoma
NY	WT	Osteosarcoma
HUO9	WT	Osteosarcoma
ZK-58	WT	Osteosarcoma
LA-N-6	WT	Neuroblastoma
SAOS-2	Null	Osteosarcoma
G-292	Null	Osteosarcoma
SKLU-1	MUT	Adenocarcinoma
CAL-78	MUT	Chondrosarcoma
CHLA-90	MUT	Neuroblastoma
SK-N-FI	MUT	Neuroblastoma
NCI-H295R	MUT	Adrenal cortical carcinoma

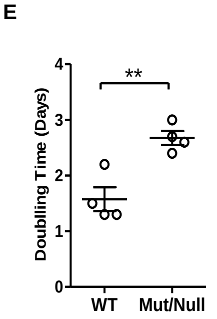

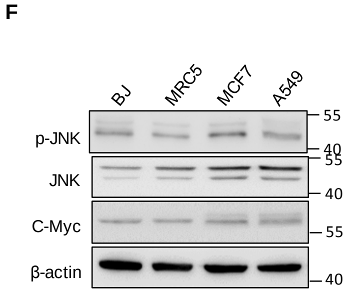

Supplementary Figure S1. DNA damage and p53 affect cell proliferation rate of ALT cells.

(A) U2OS were synchronized at G1/S by double-thymidine and released for 3, 6 and 9h, corresponding to G1, early S, middle S and late S/G2, respectively. Genome-wide and telomeric 53BP1 foci were visualized by IF-FISH. Scale bar: 10 µm.

(B) Quantification of A. Data represent the mean ± SEM, n=3. More than 100 cells were counted for each experiment.

(C) 10^6 Cells were collected and analysed by constant-field gel electrophoresis (CFGE). Genomic DNA was detected by gel-red staining, and telomeric DNA was detected by hybridization with C-rich probe.

(D) The list of all identified ALT cancer cell lines and their p53 status [1,2]. The information on p53 status is from IARC TP53 Database.

(E) The proliferation rate (doubling time) of ALT cancer cells with wt-p53 or deficient/null p53 under similar culturing conditions. Data is from published literatures [3-6]. Data represent the mean ± SEM, n=3. ** P<0.01, by 2-tailed t test.

(F) Western blot showing activation of JNK (phosphorylation) and accumulation of c-myc in BJ, MRC5, MCF7 and A549 cells.

Figure S2

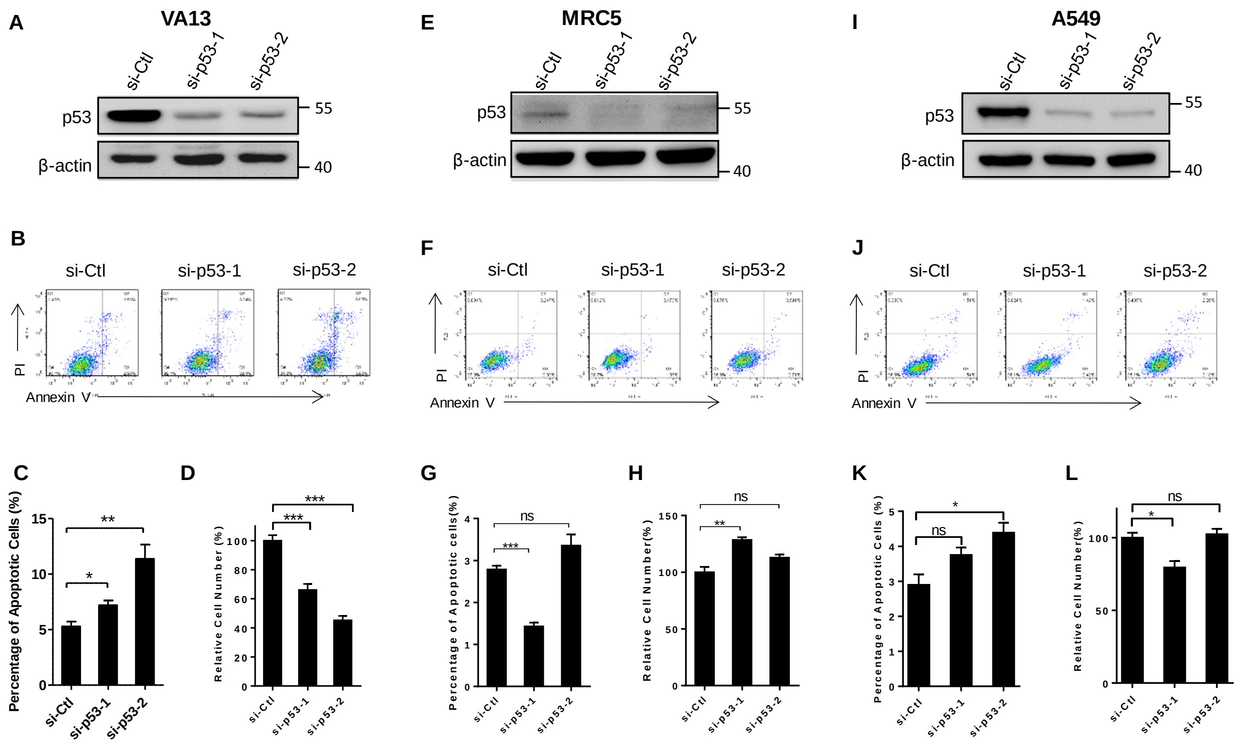

Supplementary Figure S2. The effect of p53 level on apoptosis of ALT and non-ALT cells.

(A) Western blot showing depletion of p53 in VA13 cells by siRNA.

(B) FACS analysis of apoptotic cells in control and p53-depleted VA13.

(C) Quantification of B. Data represent the mean ± SEM, n=4. * P<0.05, ** P<0.01, by 2-tailed t test.

(D) Determination of number of viable cells for VA13 with or without p53 depletion 72h after transfection. Data represent the mean ± SEM, n=6. *** P<0.0001 by 2-tailed t test.

(E) Western blot showing depletion of p53 in MRC5 cells by siRNA.

(F) FACS analysis of apoptotic cells in control and p53-depleted MRC5 cells.

(G) Quantification of F. Data represent the mean ± SEM, n=3. *** P<0.0001, by 2-tailed t test.

(H) Determination of number of viable cells for MRC5 cells with or without p53 depletion 72h after transfection. Data represent the mean ± SEM, n=3. ** P<0.01 by 2-tailed t test.

(I) Western blot showing depletion of p53 in A549 cells by siRNA.

(J) FACS analysis of apoptotic cells in control and p53-depleted A549 cells.

(K) Quantification of J. Data represent the mean ± SEM, n=3. * P<0.05, by 2-tailed t test.

(L) Determination of number of viable cells for A549 cells with or without p53 depletion 72h after transfection. Data represent the mean ± SEM, n=3. *P<0.05 by 2-tailed t test.

Figure S3

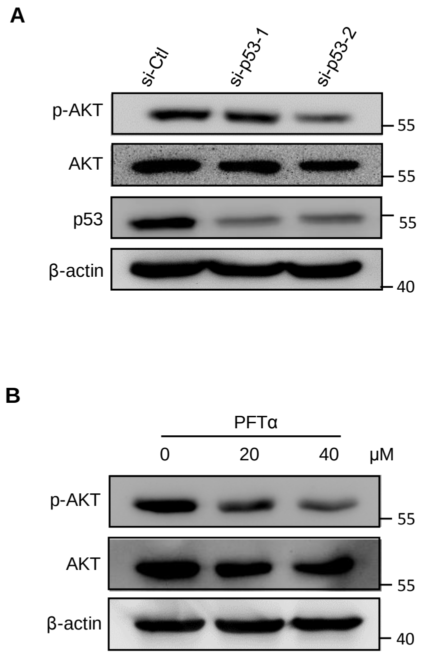

Supplementary Figure S3. P53 dependent activation of AKT in VA13 cells.

(A) Knockdown of p53 in VA13 results in decrease of p-AKT.

(B) P53 inhibitor PFTα decreases the level of p-AKT in VA13 cells.

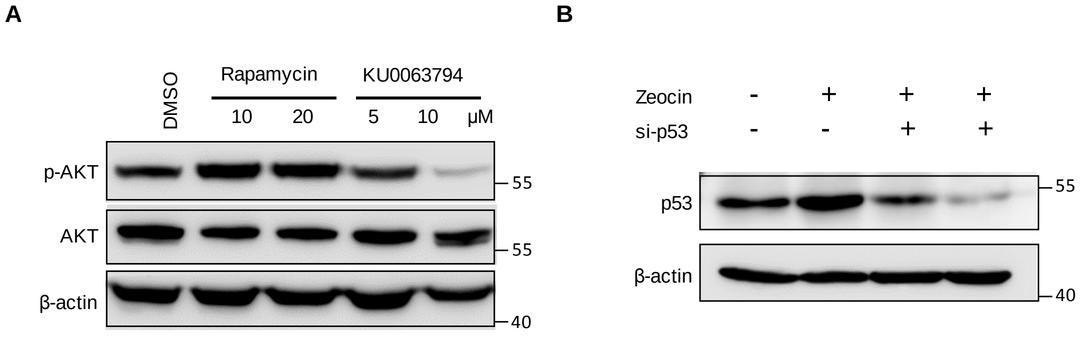

Supplementary Figure S4. mTORC2 phosphorylates AKT in U2OS cells.

(A) The phosphorylation of AKT is inhibited by mTORC1/2 inhibitor (KU0063794), but not mTORC1 inhibitor (Rapamycin).

(B) Western blot showing the knockdown of p53 in zeocin treated U2OS cells.

Figure S5

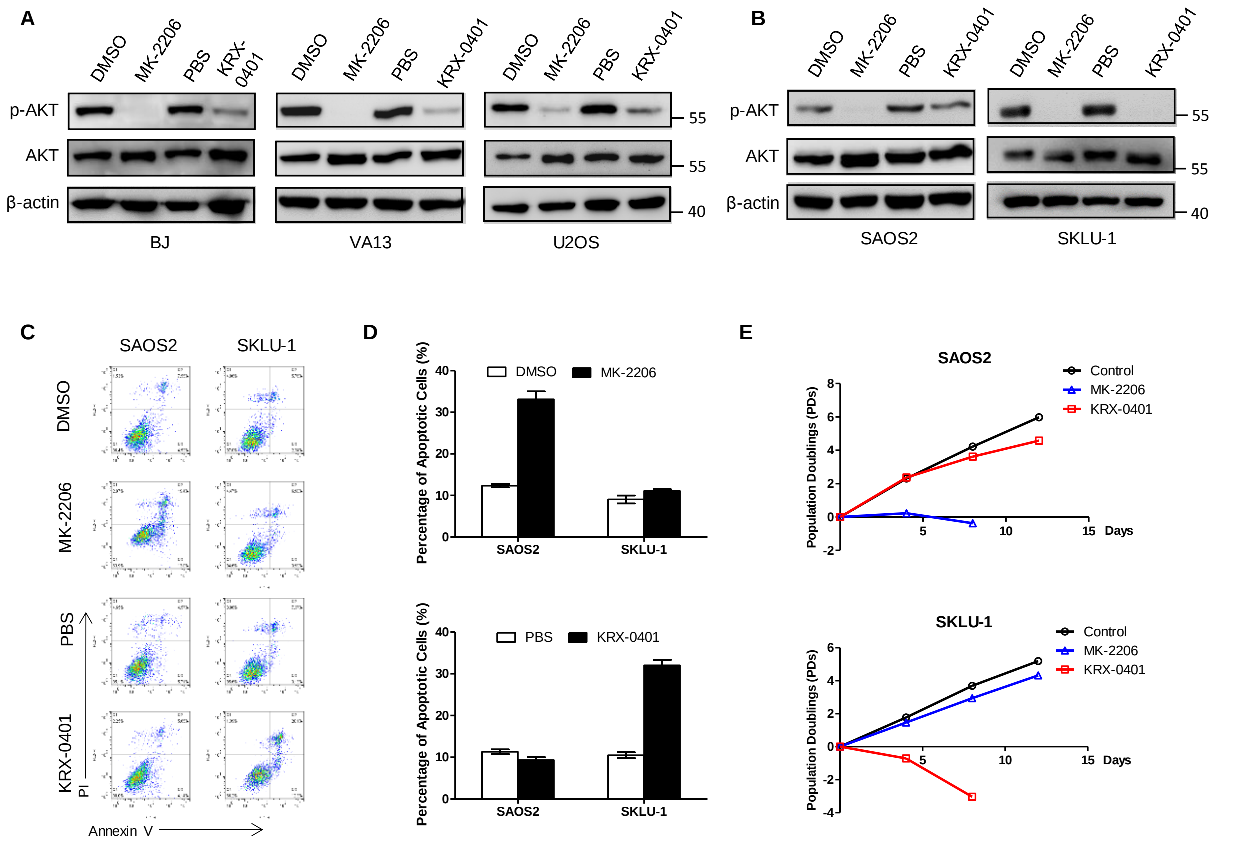

Supplementary Figure S5. AKT as a therapeutic target for ALT cancer cells.

(A) Western blot shows that AKT inhibitor MK-2206 or KRX-0401 inhibits phosphorylation of AKT in p53 positive BJ, VA13, U2OS.

(B) AKT inhibitor MK-2206 or KRX-0401 inhibits phosphorylation of AKT in p53 negative SAOS2 and SKLU-1 cells.

(C) FACS analysis of apoptosis in SAOS2 and SKLU-1 cells treated with MK-2206 or KRX-0401.

(D) Quantification of C. Data represent the mean ± SEM, n=4.

(E) Proliferation of SAOS2 or SKLU-1 cells treated with MK-2206(2.5μM) or KRX-0401 (25μM).

Figure S6

A

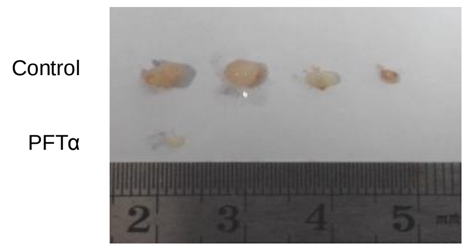

B

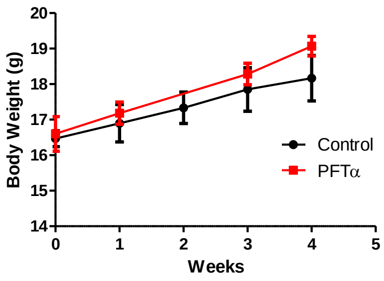

Supplementary Figure S6. The growth of ALT cancer xenograft tumors in mice treated with p53 inhibitor PFTα.

(A) Mice (female, four-week-old) were injected (i.p.) with inoculum containing 3×10^6 U2OS cells. Immediately after injection and every three days thereafter, 2.2mg/kg of PFTα dissolved in phosphate-buffered saline (PBS) was injected (Intraperitoneal injection) for 4 weeks. For the control group, the same volume of PBS was injected. Formed xenograft tumors were showed (n=4 for treated and control group).

(B) The body weight of the mice was measured over treatment period. Data represent the mean ± SEM.

Supplementary References

1 Farooqi, A. S. *et al.* Alternative lengthening of telomeres in neuroblastoma cell lines is associated with a lack of MYCN genomic amplification and with p53 pathway aberrations. *Journal of neuro-oncology* **119**, 17-26, doi:10.1007/s11060-014-1456-8 (2014).

2 Flynn, R. L. *et al.* Alternative lengthening of telomeres renders cancer cells hypersensitive to ATR inhibitors. *Science* **347**, 273-277, doi:10.1126/science.1257216 (2015).

3 Kawai, A. *et al.* Two distinct cell lines derived from a human osteosarcoma. *Journal of cancer research and clinical oncology* **115**, 531-536 (1989).

4 Cambien, B. *et al.* Silencing of hSlo potassium channels in human osteosarcoma cells promotes tumorigenesis. *Int J Cancer* **123**, 365-371, doi:10.1002/ijc.23511 (2008).

5 Lohberger, B. *et al.* Diacerein retards cell growth of chondrosarcoma cells at the G2/M cell cycle checkpoint via cyclin B1/CDK1 and CDK2 downregulation. *BMC cancer* **15**, 891, doi:10.1186/s12885-015-1915-4 (2015).

6 Lucero, C. M. *et al.* The cancer-related transcription factor Runx2 modulates cell proliferation in human osteosarcoma cell lines. *Journal of cellular physiology* **228**, 714-723, doi:10.1002/jcp.24218 (2013).

Figure S1

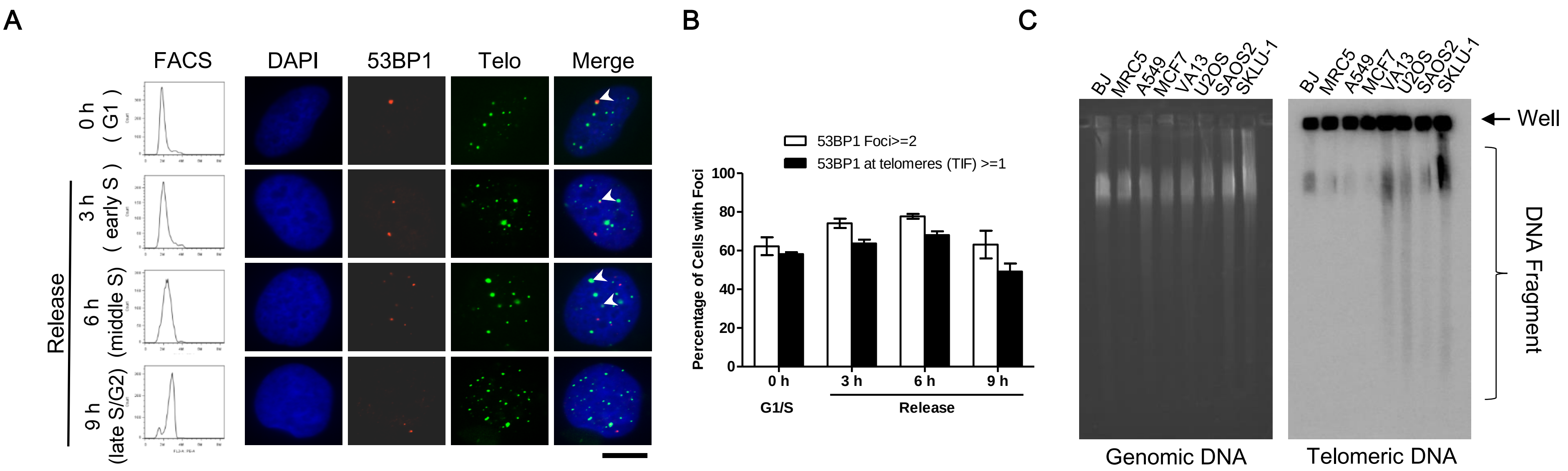

D

Cancer Cell Lines	p53	Cancer Type
U2OS	WT	Osteosarcoma
CAL-72	WT	Osteosarcoma
NY	WT	Osteosarcoma
HUO9	WT	Osteosarcoma
ZK-58	WT	Osteosarcoma
LA-N-6	WT	Neuroblastoma
SAOS-2	Null	Osteosarcoma
G-292	Null	Osteosarcoma
SKLU-1	MUT	Adenocarcinoma
CAL-78	MUT	Chondrosarcoma
CHLA-90	MUT	Neuroblastoma
SK-N-FI	MUT	Neuroblastoma
NCI-H295R	MUT	Adrenal cortical carcinoma

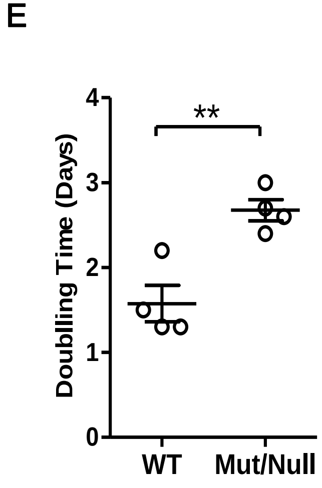

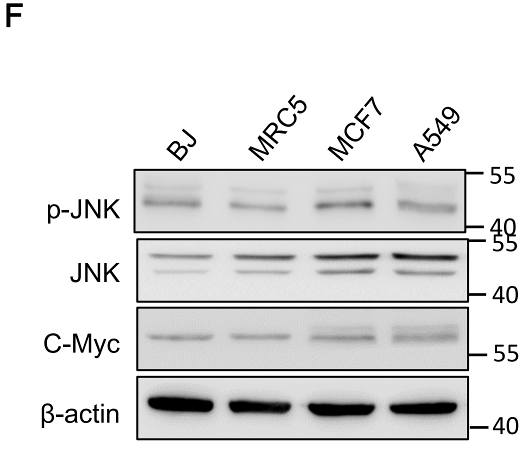

Figure S2

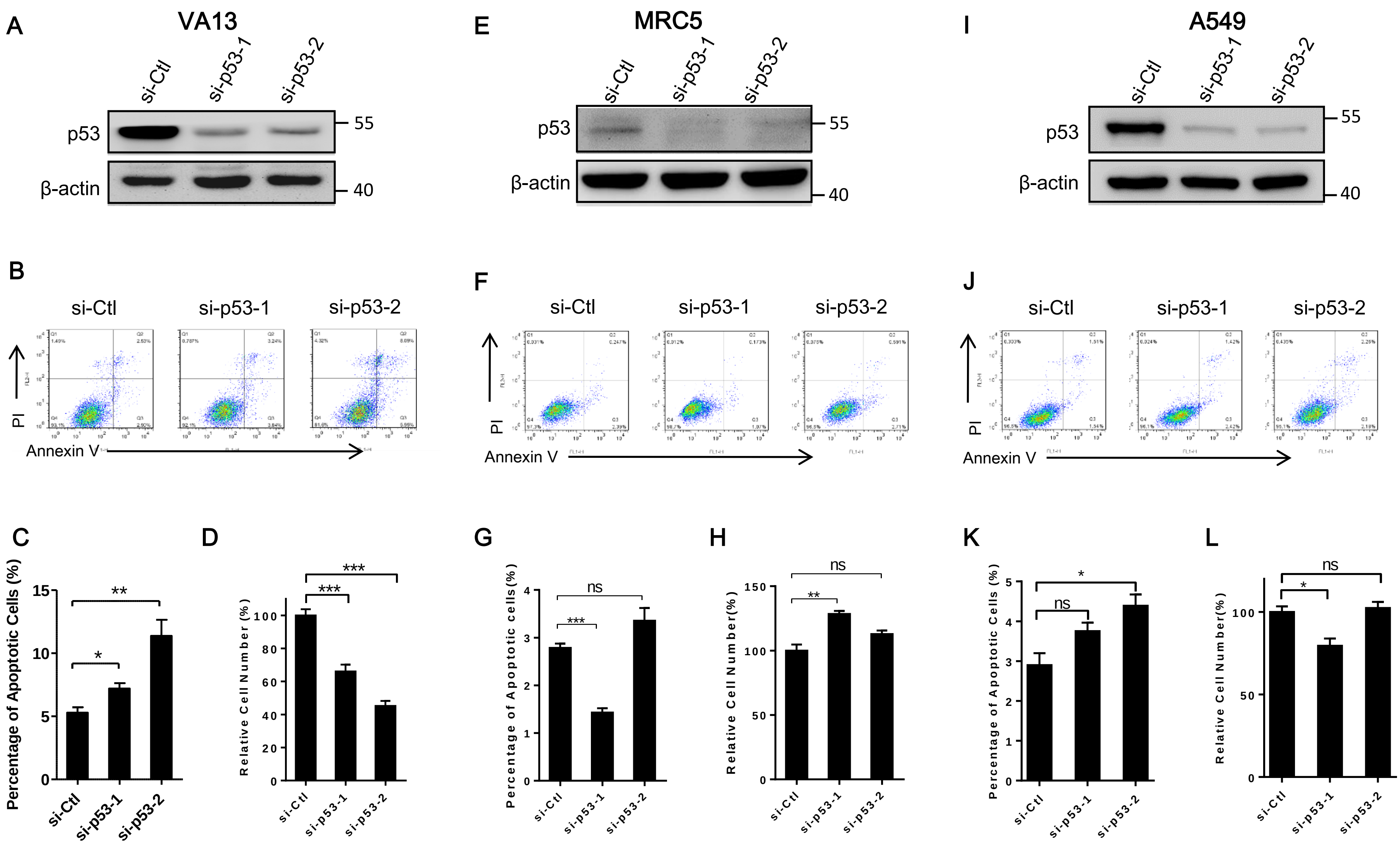

A
VA13
si-Ctl
si-p53-1
si-p53-2
p53
β-actin
55
40
E
MRC5
I
A549
B
F
J
PI
Annexin V
C
Percentage of Apoptotic Cells (%)
D
Relative Cell Number (%)
G
Percentage of Apoptotic cells(%)
H
Relative Cell Number(%)
K
Percentage of Apoptotic Cells (%)
L
Relative Cell Number(%)
ns

Figure S3

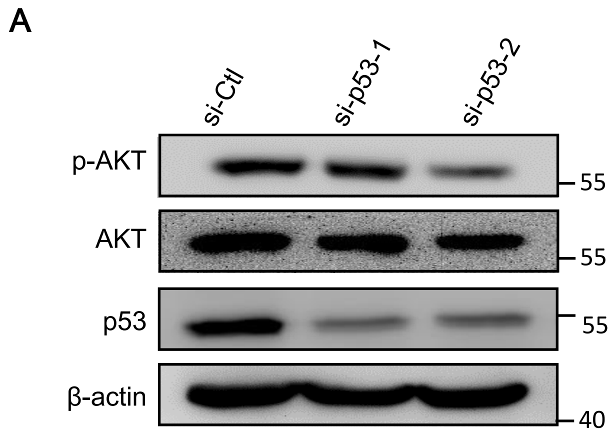
A
si-Ctl
si-p53-1
si-p53-2
p-AKT
AKT
p53
β-actin
55
55
55
40

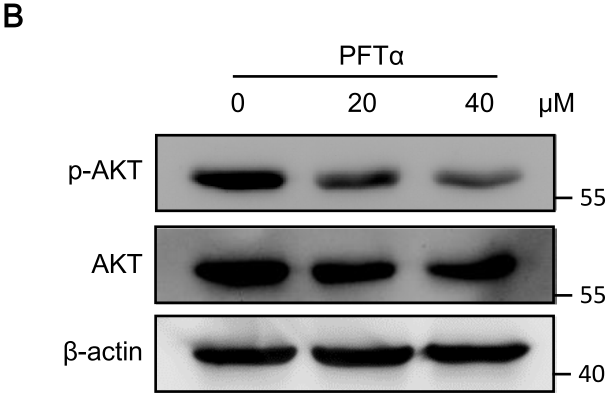
B
PFTα
0
20
40
μM
p-AKT
AKT
β-actin
55
55
40

Figure S4

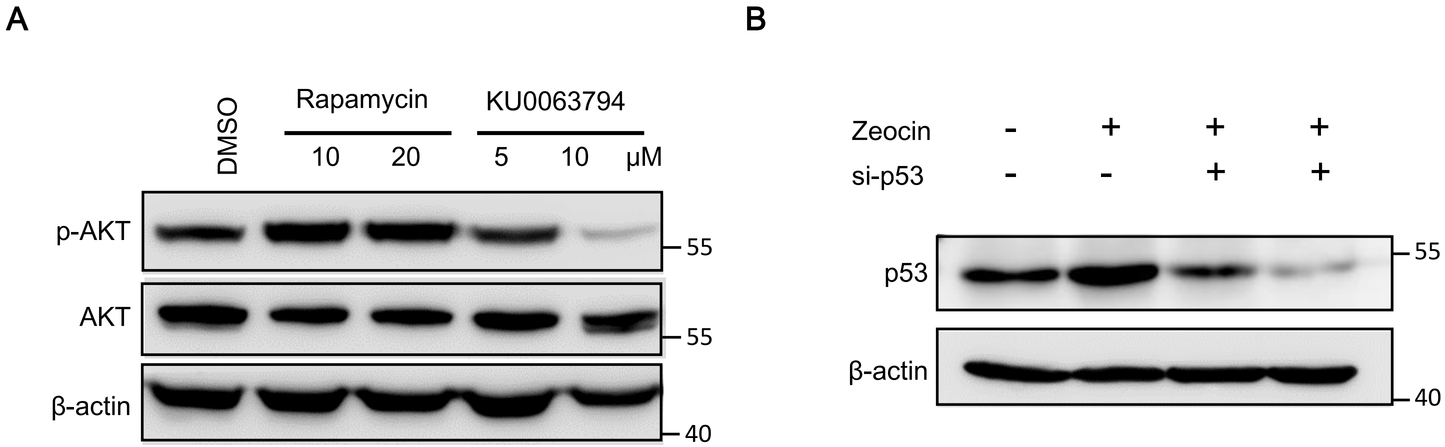
A
DMSO
Rapamycin
KU0063794
10
20
5
10
μM
p-AKT
AKT
β-actin
55
55
40
B
Zeocin
si-p53
-
+
+
+
-
-
+
+
p53
β-actin
55
40

Figure S5

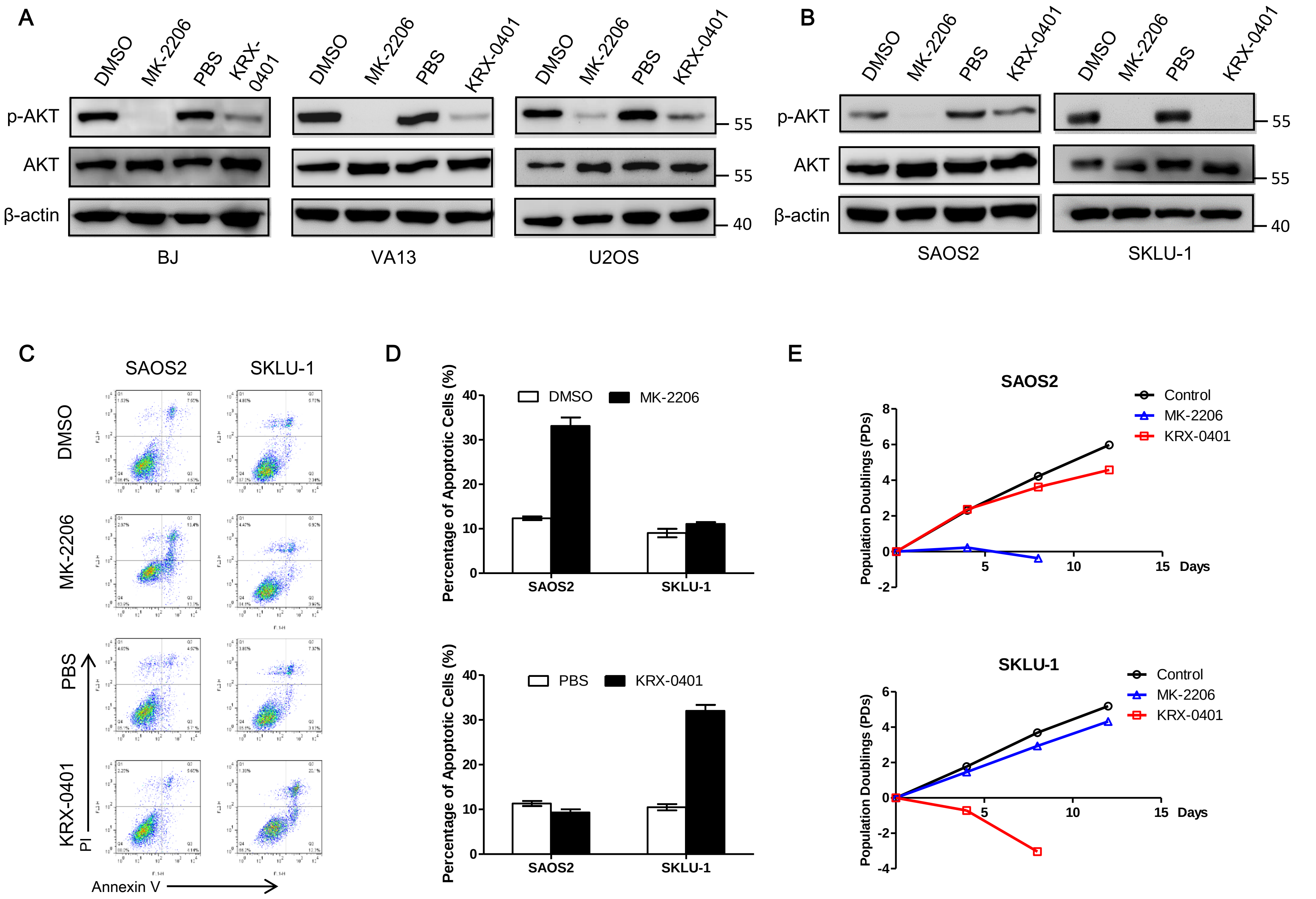
A
DMSO
MK-2206
PBS
KRX-0401
p-AKT
AKT
β-actin
55
55
40
BJ
VA13
U2OS
B
SAOS2
SKLU-1
C
SAOS2
SKLU-1
DMSO
MK-2206
PBS
KRX-0401
PI
Annexin V
D
Percentage of Apoptotic Cells (%)
DMSO
MK-2206
PBS
KRX-0401
E
SAOS2
SKLU-1
Control
MK-2206
KRX-0401
Population Doublings (PDs)
Days

Figure S6

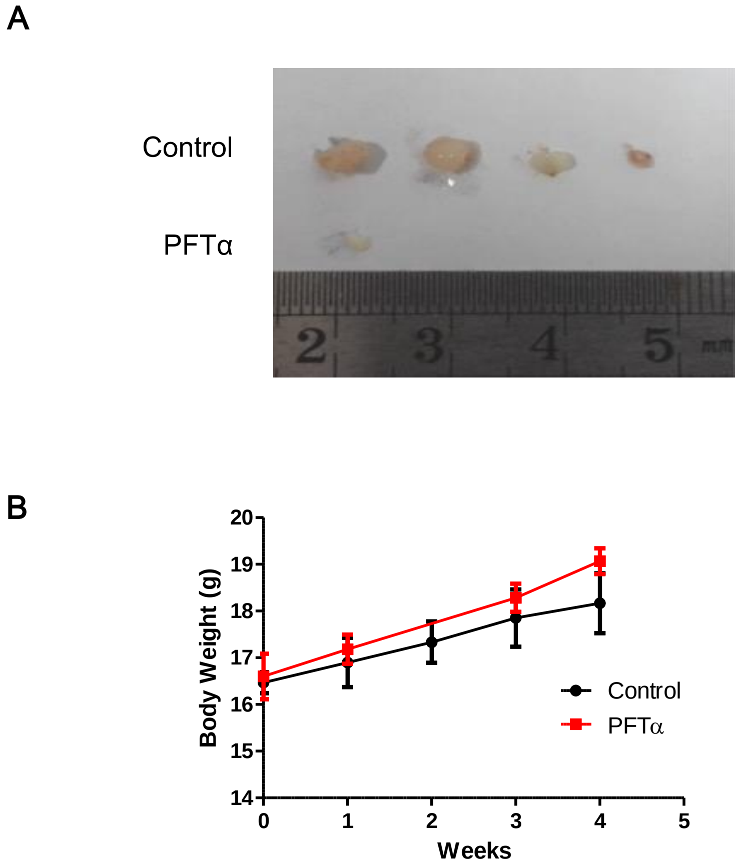
A
Control
PFTα
2
3
4
5
mm
B
Body Weight (g)
20
19
18
17
16
15
14
0
1
2
3
4
5
Weeks
Control
PFTα
